# Lentiviral silencing of GSK-3β in adult dentate gyrus impairs contextual fear memory and synaptic plasticity

**DOI:** 10.3389/fnbeh.2015.00158

**Published:** 2015-06-23

**Authors:** Benjamin Chew, Jae Ryun Ryu, Teclise Ng, Dongliang Ma, Ananya Dasgupta, Sin Hui Neo, Jing Zhao, Zhong Zhong, Zoë Bichler, Sreedharan Sajikumar, Eyleen L. K. Goh

**Affiliations:** ^1^Program in Neuroscience and Behavioral Disorders, Duke-NUS Graduate Medical SchoolSingapore, Singapore; ^2^Department of Physiology, Yong Loo Lin School of Medicine, National University of SingaporeSingapore, Singapore; ^3^Regenerative Medicine DPU, GlaxoSmithKline (China) R&D Co., Ltd.Shanghai, China; ^4^Behavioural Neuroscience Laboratory, National Neuroscience InstituteSingapore, Singapore; ^5^KK Research Center, KK Women’s and Children’s HospitalSingapore, Singapore

**Keywords:** lentivirus, GSK-3β, dentate gyrus, LTP, contextual or spatial memory

## Abstract

Attempts have been made to use glycogen synthase kinase-3 beta (GSK3β) inhibitors for prophylactic treatment of neurocognitive conditions. However the use of lithium, a non-specific inhibitor of GSK3β results in mild cognitive impairment in humans. The effects of global GSK3β inhibition or knockout on learning and memory in healthy adult mice are also inconclusive. Our study aims to better understand the role of GSK3β in learning and memory through a more regionally, targeted approach, specifically performing lentiviral-mediated knockdown of GSK3β within the dentate gyrus (DG). DG-GSK3β-silenced mice showed impaired contextual fear memory retrieval. However, cue fear memory, spatial memory, locomotor activity and anxiety levels were similar to control. These GSK3β-silenced mice also showed increased induction and maintenance of DG long-term potentiation (DG-LTP) compared to control animals. Thus, this region-specific, targeted knockdown of GSK3β in the DG provides better understanding on the role of GSK3β in learning and memory.

## Introduction

Glycogen synthase kinase 3-beta (GSK3β) is a constitutively active serine protein kinase highly expressed in the brain (Woodgett, 1990). Known targets of GSK3β number over 100, many of which are involved in pathways related to cell growth, apoptosis, metabolism, learning and memory among others (Kaidanovich-Beilin and Woodgett, 2011). GSK3β’s kinase activity is positively regulated by phosphorylation on its tyrosine 216 residue, or inhibited by phosphorylation on its serine 9 residue (Wang et al., 1994). Lithium, a known GSK3 inhibitor was demonstrated to inhibit GSK3β by increasing phosphorylation on the key serine residue (Ser9) (Jope, 2003). It was shown that administration of lithium or another GSK3 inhibitor, AR-A014418 (ARA) was able to improve cognitive performance in mouse models of neurocognitive conditions such as Down’s syndrome and Fragile X syndrome or Alzheimer’s disease (AD) respectively (Yuskaitis et al., 2010; Contestabile et al., 2013; Ly et al., 2013). GSK3 inhibition has thus been suggested as a means of prophylactic treatment for human patients with these neurological disorders (Licastro et al., 1983; Terao et al., 2006; Nunes et al., 2007; Liu and Smith, 2014). However, there are also reports showing that lithium treatment increases the risk of dementia (Dunn et al., 2005) and also resulted in impairments in cognitive performance of healthy individuals (Weingartner et al., 1985; Stip et al., 2000; Wingo et al., 2009). These inconsistent outcomes from pharmacological inhibition of GSK3 activity suggest that a more specific approach through gene targeting of selected brain regions may provide a better understanding on the function of GSK3β in cognitive functions.

Overexpression or knock-in studies in healthy mice where GSK3β is constitutively active showed spatial learning deficits (Hernández et al., 2002), impairments in novel object and inhibitory avoidance tests (Dewachter et al., 2009) and an increase in fear memory retention (Polter et al., 2010). However, results from inhibitor and transgenic knockout studies in healthy rodents are controversial. Many GSK3 inhibitors are also known to affect signaling cascades independent of GSK3 (Meijer et al., 2004). Studies administering lithium, a non-specific GSK3β inhibitor, mostly showed no effects in healthy mice (King et al., 2014). Mice administered with the GSK3β inhibitors valproic acid (Sintoni et al., 2013) and SB 216763 (Hu et al., 2009; Sintoni et al., 2013), showed impairments in the Morris water maze task and contextual fear memory consolidation but not reconsolidation in healthy mice. Heterozygous GSK3β knockout mice and mice treated with ARA showed impaired long term spatial memory and contextual fear memory reconsolidation but these mice exhibit normal contextual consolidation (Kimura et al., 2008). These discrepancies may be due to confounding factors which include toxicity from long term inhibitor administration, inhibitor non-specificity, non-site specific inhibitor effects and in the case of transgenic animals, aberrations caused by the reduction of GSK3β during brain development which are carried into adulthood.

Our study aims to further the understanding on the role of GSK3β in learning and memory and synaptic plasticity by genetically manipulating GSK3β levels specifically in the dentate gyrus (DG) of young adult mice. The DG was chosen as the target region for investigation as it is important for various forms of learning and memory, such as object discrimination and contextual fear memory (both for context memory) as well as Morris water maze (spatial memory; Rubin et al., 1999; Jeltsch et al., 2001; Lee and Kesner, 2004; Nakashiba et al., 2012).

## Materials and Methods

### Animals

All animal procedures and applicable regulations of animal welfare were performed in accordance with the GSK Policy on the Care, Welfare and Treatment of Laboratory Animals and also in accordance with Institutional Animal Care and Use Committee (IACUC) guidelines and approved by SingHealth IACUC, Singapore. Six to eight week old, male, C57BL/6 mice (of 24–28 g) were purchased through SingHealth Experimental Medicine Centre (SEMC), Singapore, and housed in Specific Pathogen Free (SPF) animal facility at Duke-NUS Graduate Medical School, Singapore. Food and water were provided to the animals *ad libitum*.

### Creation of Viral Vector

FUGW plasmid (Addgene) used for the creation of lentivirus was modified to express shRNA sequences under the control of U6 promoter and GFP under the control of human ubiquitin C promoter. Shorthairpin (shRNA) against mouse and rat GSK3β: CATGAAAGTTAGCAGAGAT (shGSK3) (Kim et al., 2006) or scrambled control sequence: TTATCAGATAGACGATTGT (shCon) (Ma et al., 2015) was cloned into the FUGW plasmid. FUGW plasmid was co-transfected with HIV-1 packaging vector Delta8.9 and the VSVG envelope glycoprotein into human embryonic kidney (HEK) 293 gp cells to produce viral particles. Growth medium was collected and subjected to ultracentrifugation at 28,000 g for 90 min to pellet viral particles. The viral pellet was re-suspended in sterile PBS and viral titre determined by infecting HEK 293 cells with serial dilutions of the virus. A viral titre of 1 × 10^10–11^ TU/ml was used for stereotaxic injections and downstream experiments.

### Western Blots

Hippocampal neurons were isolated from E18 rats as described previously (Shivaraj et al., 2012; Su et al., 2015). Dissociated neurons were cultured on poly-L-lysine coated plates and infected with lentivirus delivering shGSK3 or shCon the following day. After 5 days, cell lysate was prepared in radioimmunoprecipitation assay (RIPA) buffer (1% Triton X-100, 50 mM HEPES, pH 7.0, 150 mM NaCl, 2 mM EGTA, 0.25% sodium deoxycholate, 0.2 mM phenylmethylsulfonyl fluoride with phosphatase and protease inhibitors). For *in vivo* quantification, both hippocampi were isolated 4 weeks post stereotaxic injection and homogenized in RIPA buffer. Protein concentration was measured and 20 μg of protein was separated on an 8% SDS-PAGE gel and transferred to a polyvinylidene fluoride (PVDF) membrane. Primary antibodies used were rabbit anti-GSK3β antibody (Cell Signaling Technology, 1:5000) and Mouse anti-alpha tubulin antibody (Sigma, 1:10000). Enhanced chemiluminescence (ECL) horseradish peroxidase linked anti-rabbit or anti-mouse antibodies (GE Healthcare) were used as secondary antibodies. Restore Plus Western Blot Stripping Buffer (Thermo Scientific) was used for stripping purposes. SuperSignal West Pico Chemiluminescent Substrate (Thermo Scientific) was used to develop the blots. Histograms of all western blots were checked during the capture process by the GE LAS4000 imaging machine and also in image J. This is to ensure that all blots used for quantifications are not overexposed. Quantification of band intensities was done using image J.

### Stereotaxic Injections

Stereotaxic surgical procedures were performed under deep anesthesia (Ketamine 100 mg/ml, Xylazine 20 mg/ml) at a dose of 85 mg of Ketamine and 10 mg Xylazine per kg of animal body mass. Animals were mounted on a stereotaxic frame instruments (Kopf Instruments, Tujunga, CA, USA). An incision was made along the midline of the scalp and the skull exposed. Small burr holes were drilled into the skull at the following coordinates as previously described (Ge et al., 2006; Zhao et al., 2015): (1) 2 mm posterior to bregma, ±1.6 mm lateral to midline, 2.5 mm ventral from skull; (2) 3 mm posterior to bregma, ±2.6 mm lateral to midline, 3.2 mm ventral from skull. Lentivirus was injected using a 1 μl Hamilton syringe at a volume of 0.5 μl per site (flow rate of 0.05 μl/15 s). 0.5% Bupivacaine was administered after the surgery to provide acute pain relief. 1–5 mg/kg of Butorphanol was administered subcutaneously for 2 days after surgery to relief pain from the surgical procedure and to ensure that animals experience little or no discomfort after the surgery. Animals showing signs of pain and/or obvious discomfort outside this time period were removed from the study and euthanized.

### Electrophysiology

Hippocampal slices of 12 wild type mice of 10–12 weeks of age injected at 6–8 weeks old with shCon (six mice) or shGSK-3β (six mice) were used (4 weeks after injection) for electrophysiological recordings as previously described (Sajikumar et al., 2005). Briefly after anesthetization using CO2, mice were decapitated and the brains were quickly removed and cooled in 4°C artificial cerebrospinal fluid (ACSF). Transverse hippocampal slices (400 μm) were prepared from the right hippocampus using a manual tissue chopper and the slices were incubated at 32°C in an interface chamber. The ACSF contained the following (in mM): 124 NaCl, 4.9 KCl, 1.2 KH_2_PO_4_, 2.0 MgSO_4_, 2.0 CaCl_2_, 24.6 NaHCO_3_, 10 D-glucose, equilibrated with 95% O_2_–5% CO_2_ (32 L/h). Slices were preincubated for 2.5 h. Recordings in the DG were performed similar to that method described in Walther et al. (1998) and Balschun et al. (1999). After the preincubation period, a monopolar lacquer-coated, stainless-steel electrode (5 MΩ; AM Systems, United States of America) was placed in the stratum moleculare of the DG to stimulate the medial performant path input. About 200 μm apart, the recording electrode was lowered to the same level to record field excitatory postsynaptic potentials (fEPSPs). The stimulation strength was adjusted to elicit a fEPSP slope of 40% of the maximum of the corresponding I/O curve. Long-term potentiation (LTP) was induced by a repeated, 3-fold tetanization paradigm consisting of 15 bursts of eight pulses, 200 Hz, interburst interval 200 ms, which were applied with an interval of 10 min. The slopes of the fEPSPs were monitored and the baseline was recorded for 30 min before LTP induction. Four 0.2-Hz biphasic constant-current pulses (0.1 ms/polarity) were used for baseline recording.

### Behavioral Tests

All behavioral tests were performed in an individual, dedicated experimental room. On the testing day, animals were put in the test room at least 20 min before testing in order to acclimatize. The experimenter was always blind to the treatment type when performing the tests.

### Open Field Activity

Mice were placed in a 40 × 40 × 40 cm transparent Plexiglas arena and allowed to explore freely for 10 min. Exploratory activity was recorded with a TopScan behavior monitoring and analysis system (CleverSys). Exploratory behavior in the middle of the arena was determined by splitting the arena into 4 × 4 squares within the software and considering the middle 2 × 2 square as the middle of the arena.

### Object Location Test

The experiment was performed and data analyzed as previously described (Barker and Warburton, 2011). For the first 2 days, mice were allowed to explore and habituate in a 40 × 40 × 40 cm transparent Plexiglas arena for 10 min each day. On the third day, mice were trained by placing them in the same arena with two similar objects at different corners of the arena for 10 min. After 24 h, the mice were placed in the same arena with identical objects, with one of the object at a novel location and another at a familiar location. Exploratory behavior was recorded with a TopScan behavior monitoring and analysis system (CleverSys) for 10 min and the amount of time a mouse spends exploring an object was timed by hand. Object exploration was defined as a nose poke within a 1 cm of the object. Object location discrimination ratio was calculated by subtracting familiar object exploration time from novel object exploration time and dividing the difference by the total time spent exploring both objects.

### Morris Water Maze

The water maze consisted of a 120 cm diameter gray circular pool filled with water (23–26°C, 40 cm deep) made opaque by adding non-toxic white paint (Crayola®). The pool was surrounded by several distant cues from the environment of the experimental room. The animals learned to find a transparent platform (10 cm in diameter) hidden 1 cm below the water surface, and which location remained constant throughout the experiment. Each animal was tested four times a day at five consecutive days, with an inter-trial of about 20 min. For each day, mice were systematically weighted before the first trial then released facing the tank wall from one randomly selected starting points (North, South, East or West), and allowed to swim until they reached the platform. Animals that failed to find the platform within 60 s were gently directed to it and put on it for 15 s. After the trial, mice were removed from the pool, gently dried with a towel and placed individually in cage filled with paper towel and warmed with water bottles placed under the cages to avoid hypothermia. The criterion of learning success consisted to reach the platform in less than 20 s. On the 6th day, a 60 s probe trial was conducted without platform and the time spent in each quadrant was analyzed. This trial was followed by a 60 s cued test to check the visual acuity of the mice. Trials were recorded with a video camera placed above the center of the pool and the quantitative analysis was automated by means of the ANY-maze® video tracking system (Stoelting, USA).

### Fear Conditioning

All fear training was performed in a set of four identical fear-conditioning chambers (Med Associates), equipped with a Med Associates Video Freeze system. Individual boxes were enclosed in sound-attenuating chambers (Med Associates). The grid floor consisted of stainless steel rods. Chambers were individually lit from above with white house lights and cleaned with 70% isopropyl alcohol in between squads. On the day of training mice were placed into individual experimental chambers set up to conditioning context A which consisted of a bare chamber with white walls, steel rod floor, undiffused white house lights, and lightly scented with Septanol (ICM Pharma). Following a 180 s baseline period of exposure to the context A, mice were fear conditioned using a 30 s, 5000 Hz, 90 dB tone co-terminating with a 0.7 mA, 2 s foot shock. Following the shock, mice were given 120 additional seconds in the same context before being removed. Contextual fear memory retrieval was assessed 24 h later by placing mice back into the same context for a 5 min exposure session. Cue fear memory retrieval was performed 1 h after contextual fear retrieval. The environment was changed to context B consisting of a smooth white floor, diffused white lighting, walls of a different texture and color, and the chamber scented with a different scent. Mice were exposed to the new context for 120 s before being exposed to a 30 s, 5000 Hz, 90 dB tone. Mice were further monitored for freezing activity for another 4.5 min before being returned to their home cages. For fear acquisition, average freezing after foot shock administration was scored and analyzed. For contextual memory test, freezing across the first 3 min of context exposure A was scored. For cue fear memory, freezing activity in the context B for 5 min from the presentation of the tone was scored and analyzed.

### Perfusion and Sectioning

Mice were deeply anesthetized with pentobarbital and transcardially perfused with chilled physiological saline followed by 4% paraformaldehyde in PBS. Extracted mice brains were post fixed in the same buffer for 8 h before being transferred to 30% sucrose solution and stored at 4°C. Fixed mice brains were sliced with a sliding microtome (Leica) to obtain 40 μm-thick coronal sections. Slices containing hippocampus were collected at every 6th interval in an anterior to posterior manner and used for downstream experiments.

### 5-ethynyl-2′-deoxyuridine (EdU) Cell Counting within the DG

Animals were injected with 100 μg of EdU on the day of lentiviral injection. Animals were sacrificed after 28 days. Brain sections were stained for EdU with Click-iT EdU Alexa Fluor 647 kit (Invitrogen) and 4′,6-diamidino-2-phenylindole (DAPI; Invitrogen) according to manufacturer’s instructions. Sections were imaged with a Zeiss LSM710 Laser scanning confocal microscope. Total number of EdU positive cells within the granular layer and the area of the granular layer was quantified for each section. Granular layer volume is calculated by multiplying the total area by 40 μm which is the thickness of each section and by 6 which is the sectioning interval between each collected section. Total density of EdU positive cells in the granular layer was calculated by dividing the total EdU cell number by volume of the granular layer.

### Immunohistochemistry

The following primary antibodies were used to immuno-stain brain sections: goat anti-doublecortin (Santa Cruz, 1:500), mouse anti-NeuN (Milipore, clone A60, 1:500), rabbit anti-GSK3β (Cell Signaling Technology, 1:200). Brain sections were blocked in 5% donkey serum in tris-buffered saline (TBS) and 0.1% Triton-X for 1 h. Brain sections were incubated with primary antibodies at 4°C overnight. Appropriate Alexa Fluor 555 or 647 antibodies (Invitrogen, 1:500) were incubated with the brain slices for 2 h at room temperature. Further staining with Click-it EdU Alexa Fluor 647 kit (invitrogen) and DAPI (Invitrogen) was performed if needed. Brain sections were then cover slipped and kept at 4°C in a light proof box.

### Statistics

Data were statistically analyzed using Student’s-*T*-test unless otherwise stated. The average values of the slope function of the field EPSP (in millivolts per millisecond) per time point were subjected to statistical analysis using the Wilcoxon signed rank test when compared within one group; *p* < 0.05 was considered as statistically significant different.

## Results

### Lentivirus Delivers shRNA to Cells in the Granular Layer and Hilus of the DG

**Figure 1:**
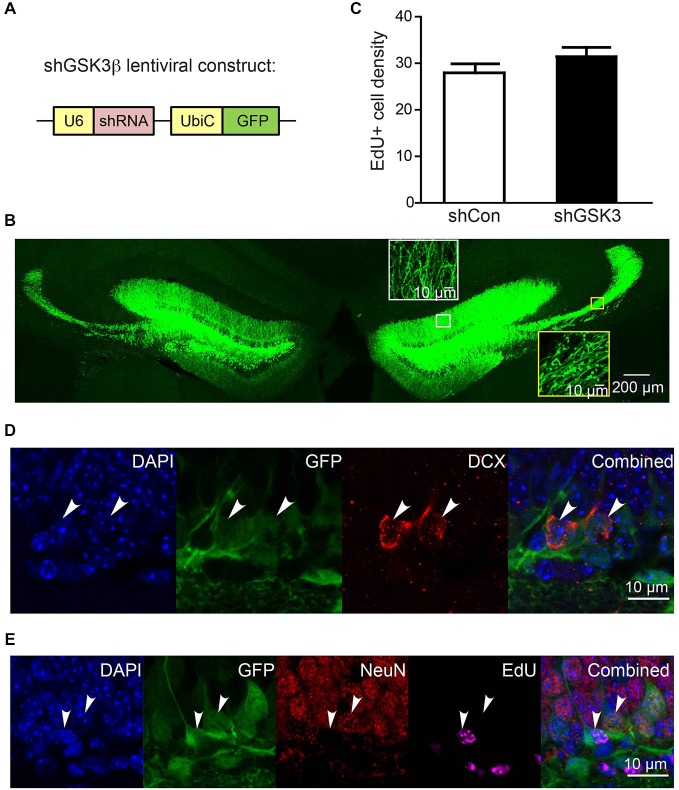
**Efficiency of lentivirus infection in dentate gyrus (DG) of injected brains. (A)** Schematic diagram showing knockdown (KD) lentiviral construct with shRNA expression driven by U6 promoter and GFP expression driven by ubiquitin C promoter. **(B)** Representative image showing typical infection efficiency of KD lentivirus in DG in both sides of the brain. Insets in white box and yellow box are high magnification images of dendrites and axons of lentivirus-infected cells, demonstrating dendrites projecting to molecular layer and axons projecting through mossy fibers to CA3 region respectively. **(C)** Graph showing number of EdU+ cells per area (108 μm^3^) of DG granular cell tissue at 28 days post injection (*n* = 3 animals per group) (error bars shown represented SEM). **(D,E)** Representative images showing GFP expression in granular cells co-expressing DCX (immature neuronal marker), NeuN (mature neuronal marker) and cells labeled with EdU (proliferating cells) at 10 days post injection. EdU was injected on the same day as virus injection. Scale bar = 200 μm for **(B)** or 10 μm for inset in **(B)** and for **(D,E)**.

We first examined the delivery efficiency of lentivirus carrying shRNA constructs by injecting lentivirus constructs coding for shRNA against either GSK3β (shGSK3) or shRNA with scrambled sequence (shCon) (Figure 1A) into the DG of young adult mice. Green fluorescence protein (GFP) expression was detected in cells within the granular layer and the hilus of the DG at both sides of the brains 28 days post injection (Figure 1B). Axons and dendrites from GFP labeled cells within the granular layer are clearly visible and extend towards the CA3 region through mossy fiber and towards molecular layer respectively (Figure 1B).

Previous literature has suggested that the inhibition of GSK3β is able to promote neurogenesis *in vitro* and *in vivo* (Morales-Garcia et al., 2012). To explore if neurogenesis was impacted in our knockdown model, EdU was injected on the same day as lentiviral injections. A quantification of EdU-positive cells within the DG granular layer showed no statistically significant difference between shCon and shGSK3 groups (Figure 1C). DG GSK3β silencing in our mice model thus, has no significant impact on neural progenitor cell proliferation within the DG. We next determined if our lentivirus has a preference to infect progenitor cells or granular layer neurons in immature or mature stages of development. Fluorescence imaging on brain slices from mice 10 days post injection showed that subpopulations of GFP expressing cells for both constructs expressed the immature neuronal marker doublecortin (DCX; Figure 1D), or the mature neuronal marker NeuN (Figure 1D), and can be labeled with EdU (Figure 1E). We show here that there are many NeuN positive cells, lesser DCX positive cells and very few EdU infected cells, which corresponds to the typical cell composition in the DG. Our lentivirus is thus able to infect and deliver short hairpin constructs into dividing cells and neurons at various phases of development without any significant preferences for any specific cell types.

### shRNA against GSK3β Reduced GSK3β Protein Levels but has no Impact on Hippocampal Neurogenesis

**Figure 2:**
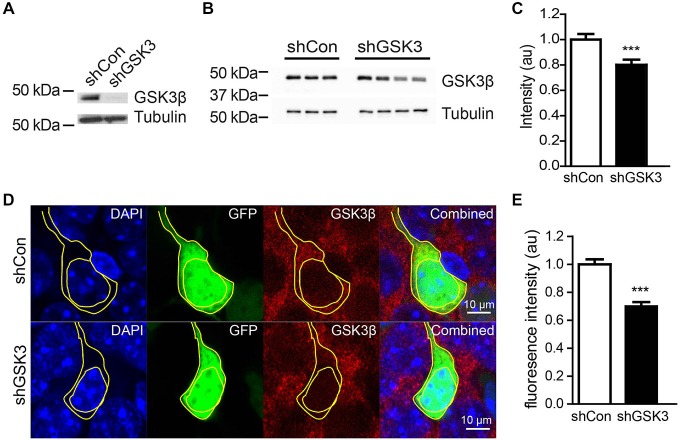
**Efficiency of lentivirus carrying shRNA in knocking down GSK3β. (A)** Representative western blot of GSK3β in primary cortical neurons infected with lentivirus expressing shCon or shGSK. **(B)** Representative western blot of GSK3β from whole hippocampal extracts of three shCon and four shGSK3-injected mice. **(C)** Quantification of GSK3β protein levels from whole hippocampal extracts of shCon and shGSK3-injected mice. **(D)** Representative images showing immunostaining against GSK3β in shCon or shGSK3 expressing cells in DG. **(E)** Graph showing quantification of GSK3β fluorescence intensity in the cytoplasm of cells expressing shGSK3β as compared to those expressing shCon. Error bars shown represented SEM. Student’s-*T*-test ***P < 0.001, Scale bar = 10 μm.

We next sought to determine the efficiency of our shGSKβ in reducing GSK3β protein levels *in vitro* and *in vivo*. Lentiviruses carrying shCon or shGSK3 were used to infect cultured rat primary cortical neurons. shGSK3, but not shCon was able to reduce the expression of GSK3β in these cultured neurons (Figure 2A). To validate the knockdown of GSK3β *in vivo*, mice injected with virus were sacrificed 28 days post lentiviral injections. Whole hippocampal extracts showed significantly lower levels of GSK3β (Figures 2B,C), *P* = 0.0003. To further verify the efficiency of GSK3β knockdown in individual cells in the DG, brains were sectioned and immuno-stained for GSK3β. Cells infected with lentivirus expressing shGSK3 showed lower levels of GSK3β fluorescence intensity in the cytoplasm as compared to those expressing shCon (Figures 2D,E). Thus, our shGSK3 construct efficiently reduced GSK3β protein levels both *in vitro* and *in vivo*.

### Silencing of GSK3β in the DG has no Significant Effect on Locomotor Activity or Anxiety-Like Behavior

**Figure 3:**
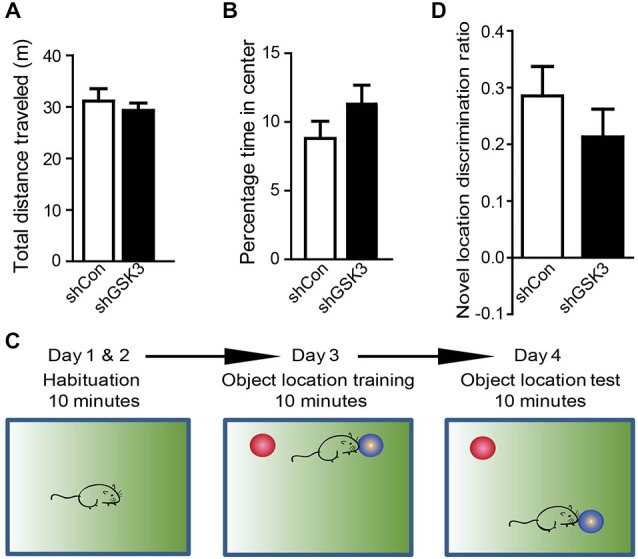
**Silencing of GSK3β in DG does not affect locomotor activity, anxiety-like behavior and object discrimination. (A)** Graph shows open field activity of shCon or shGSK3β lentivirus-injected mice. (shCon: *n* = 12, shGSK3: *n* = 15 mice) **(B)** Graph shows amount of time spent by both groups of mice in the middle of the open field arena. **(C)** Illustrations showing object location training and test procedure. **(D)** Graph shows object location discrimination between both groups of mice. (shCon: *n* = 12, shGSK3: *n* = 9 mice, error bars are SEM).

GSK3β has been implicated in hyperactivity and anxiety (Polter et al., 2010). Differences in locomotor activity or anxiety between mice groups may confound results in behavioral tests that assess learning and memory. To determine if there are differences in locomotor or anxiety-like behavior levels between shCon and shGSK3 mice, mice were monitored in an open field 4 weeks after injection (28 DPI). No significant differences in distance traveled were found between groups (Figure 3A). The percentage time whereby a mouse spends in the middle of the open field has been used as a measure of anxiety (Ramboz et al., 1998). Both groups showed similar times spent in the middle of the open field. (Figure 3B) Silencing of GSK3β in the DG therefore did not significantly impact locomotor activity and anxiety-like behavior.

### Silencing of GSK3β in the DG has no Significant Effect on Acquisition and Retrieval of Long-term Spatial Memory

To assess if silencing of GSK3β in the DG will affect spatial learning and memory in healthy mice, two hippocampus-dependent spatial memory tasks were used, namely the spatial object location task (experimental design summarized in Figure 3C) and the Morris Water Maze. During the test phase of the spatial object location task, both groups of mice spent more time exploring the object in the novel location as represented by the positive discrimination ratios. The discrimination ratios in our study were in line with other studies showing learning in the object location task (Ennaceur et al., 1997; Kesby et al., 2015). However there were no significant differences between the discrimination ratios of both control and GSK3β-silenced mice (Figure 3D).

**Figure 4:**
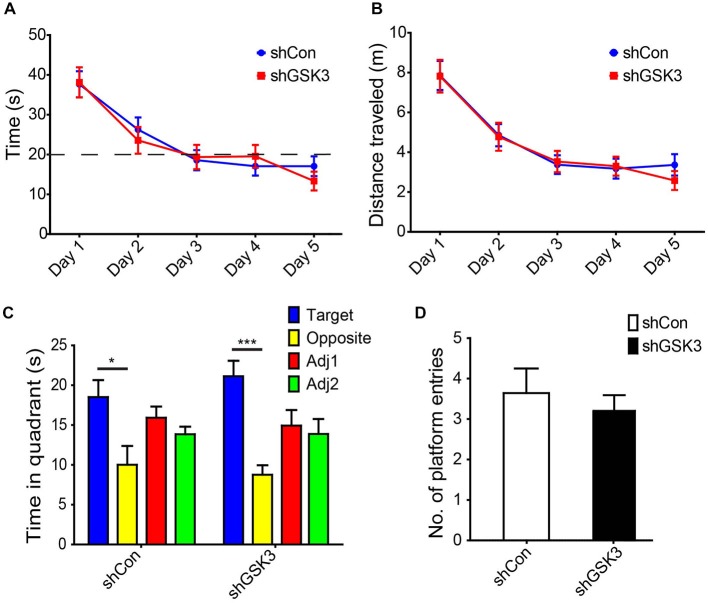
**Knocking down of GSK3β does not affect spatial memory in Morris water maze. (A)** Graph shows average time needed for both shCon or shGSK3β lentivirus-injected mice to find hidden platform for each day of training (with four trials per day pooled). **(B)** Graph shows average distance swum by each group of mice to find hidden platform for each day of training. **(C)** Graph shows time spent exploring each quadrant during the probe trial (shCon: *n* = 11 shGSK3: *n* = 10, *p < 0.05, ***p < 0.001, error bars are SEM, ANOVA followed by Bonferroni *Post hoc* comparisons). **(D)** Graphs shows number of entries into platform area by both groups of mice during the probe trial.

To validate our object location test results, a Morris water maze-based analysis was performed. With repeated training, both groups showed a similar decrease in time and swim distance required to locate the hidden platform (Figures 4A,B). During the probe trial, both groups spent significantly more time in the quadrant where the platform was previously located as compared to the opposite quadrant (Figure 4C). The number of entries in the area where the platform was similar in both groups as well (Figure 4D). Together these results suggest that spatial learning and memory is not significantly affected in our DG GSK3β-silenced mice.

### GSK3β Silencing Impairs Contextual Fear Memory

**Figure 5:**
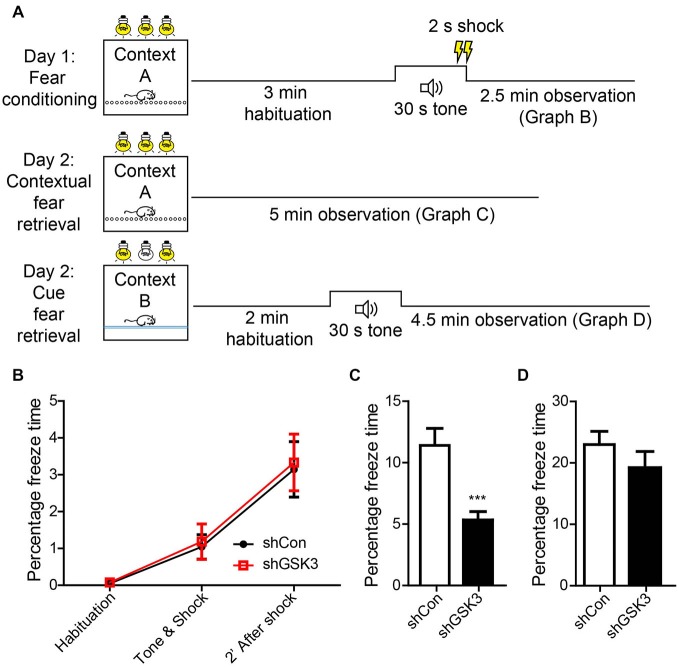
**Knocking down of GSK3β affects contextual memeory. (A)** Schematic diagram shows habituation and test procedures for contextual fear memory test. **(B)** Graph shows percentage of freezing time during various phases of fear training. **(C)** Graph shows percentage of time freezing across 3 min when mice were placed back in the fear chamber 24 h after training (Student’s-*T*-test ***p < 0.001). **(D)** Graphs shows percentage of time freezing when mice were placed in a contextually different chamber but presented with an identical tone (shCon: *n* = 25, shGSK3: *n* = 14, error bars SEM).

In addition to spatial memory, the hippocampus is also involved in contextual memory consolidation. To assess if GSK3β silencing has an effect on contextual memory, we examined mice using a contextual fear-conditioning task (Figure 5A). During the habituation phase of training, both groups showed negligible freezing times. After exposure to a 30 s tone and 2 s shock, both groups showed significantly increased freezing indicating the acquisition of contextual fear memory (Figure 5B). After 24 h, both groups were assessed for the retrieval of contextual fear memory. The GSK3β-silenced group showed significantly lower freezing times as compared to control (*p* = 0.0004), indicating an impairment in contextual fear memory (Figure 5C). As the amygdala is well known to contribute towards contextual fear memory, we performed an amygdala-dependent, hippocampus-independent cued fear memory test to assess amygdala function. After exposure to the cue in a novel environment, both groups of mice showed similar levels of increased freezing (Figure 5D). The impairment in contextual fear memory formation observed in DG GSK3β silenced mice was therefore due to defective consolidation by the hippocampus.

### GSK3β Silencing Increases LTP

**Figure 6:**
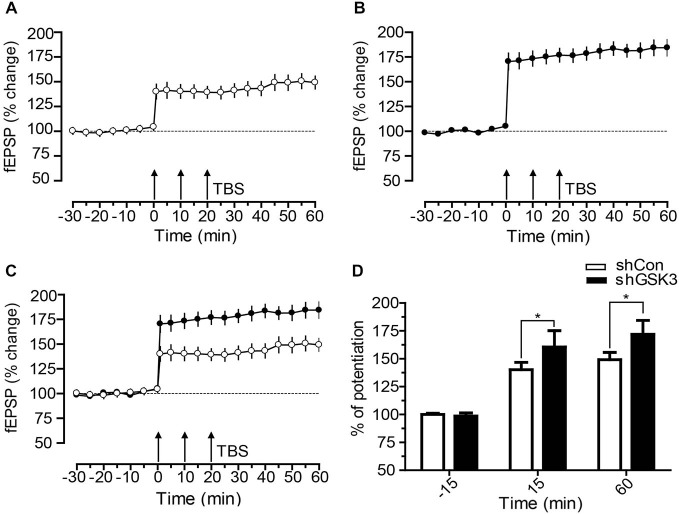
**Time course of DG-LTP in mice expressing shCon or shGSK3 in DG. (A,B)** After recording a stable baseline of 30 min, three times TBS was applied within an interval of 10 min (3 × TBS) which resulted in lasting potentiation up to 60 min. shGSK3 group (n = 12) **(B)** showed higher potentiation compared to control group (n = 11) **(A)**. **(C)** Graph shows merged control and shGSK3 LTP for direct comparison. **(D)** The bar graph represents the difference in the percentage of potentiation at −15, 15 and at 60 min after the induction of LTP in control (open bar) and shGSK3 (filled bar). The asterisks in 15 and 60 min represent statistically significant potentiation (Mann Whitney *U* Test *p < 0.005) in shGSK3 group as compared to the shCon group. Triple arrow represents TBS applied for inducing LTP. Error bars represent SEM.

To investigate if the contextual fear memory impairment observed in shGSK3 mice may be due to changes in synaptic plasticity, we performed LTP recordings on brain slices from naïve mice harvested 28 days post lentiviral injection. LTP was induced in both groups, and both groups showed maintenance of late LTP (Figures 6A,B). However, shGSK3 mice showed a much higher level of LTP upon induction as compared to control. This difference in LTP level between the groups persisted throughout the recording period of 60 min. Figure 6A showed statistically significant potentiation compared to its own base line (−15 min) after LTP induction (+15 min and +60 min, *p* = 0.0010 in both cases, Wilcoxon Sign Rank test). Similarly, post tetanic potentials at +15 min and +60 min in Figure 6B was also significantly higher compared to its own baseline (*p* = 0.0005 in both cases, Wilcoxon Sign Rank test). A comparison between the LTP from DG of control and shGSK3 mice (Figures 6C,D) also revealed statistically significant potentiation in DG-LTP in shGSK3 compared to control mice (*p* = 0.0005 and *p* = 0.0006 respectively at +15 and +60 min, Mann Whitney *U* Test).

## Discussion

Previous studies examining the cognitive effects of GSK3β manipulation have typically used transgenic or pharmacological inhibitor animal models. However, functional dissection of a multi-functional protein kinase such as GSK3β with differing roles in the developing and adult brain (Salcedo-Tello et al., 2011) or in different tissues in the body (Patel et al., 2008) will require a more targeted approach. To our knowledge, our study is the first to assess the cognitive effects of long term GSK3β silencing in the DG of mice during young adulthood.

Long term gene knockdown with the use of lentiviral vectors delivering shRNA has been successfully performed previously (Abbas-Terki et al., 2002). Our lentiviral-mediated delivery of shRNA against GSK3β into the mouse DG was to reduce GSK3β levels within DG granule cells. In our study, whole hippocampal GSK3β protein levels showed a modest but significant reduction of GSK3β. This modest reduction is likely due to diluting effects from the surrounding non-transduced tissue within and around the DG. Nonetheless, our knockdown was sufficient to elicit behavioral and electrophysiological differences between both groups of animals.

Cells infected by our lentivirus included dividing cells and neurons at various stages of maturation; both of which are known to play important roles in hippocampus-dependent learning and memory (Saxe et al., 2006; Deng et al., 2009; Gu et al., 2012; Nakashiba et al., 2012; Vukovic et al., 2013).

Overexpression or knock-in studies where GSK3β activity is significantly higher than physiological levels have resulted in decreased neurogenesis (Eom and Jope, 2009; Sirerol-Piquer et al., 2011). In neurocognitive disorders such as Down syndrome and Fragile X where neurogenesis is impaired, administration of GSK3β inhibitors were shown to enhance neurogenesis (Guo et al., 2012; Contestabile et al., 2013). The effects of GSK3β inhibition on neurogenesis in healthy adult mice are not as clear. Several studies utilizing various GSK3β inhibitors showed an increase in adult neurogenesis (Boku et al., 2009; Eom and Jope, 2009; Morales-Garcia et al., 2012), while a study using valproic acid (Sintoni et al., 2013) did not find any significant changes. The differences in observations may be due to differences between drugs, dosage and types of cells or animal models used. We found that shRNA-mediated silencing of GSK3β did not significantly change cell proliferation levels within the DG, suggesting that it is unlikely that the behavioral phenotypes we observed are due to the potential effect of cell-autonomous GSK3β silencing on cell proliferation.

We found silencing of GSK3β in the DG particularly impacted long-term contextual fear memory retrieval but not that of long-term spatial memory. Although the DG is involved in both contextual and spatial memory, both types of memory have been shown to involve different molecular and neural pathways (Bach et al., 1995; Mizuno and Giese, 2005). Contextual fear memory has been shown to be disrupted by lesions of the entorhinal cortex while spatial memory was not affected (Burwell et al., 2004). The DG receives inputs from the entorhinal cortex via the perforant path (Lomo, 1971) and overexpression of GSK3β impairs LTP recorded from the CA3 or DG upon stimulation of the perforant path (Hooper et al., 2007; Zhu et al., 2007). Therefore, it is possible that GSK3β in the DG may have a role in the processing of inputs from the perforant pathway. The lack of spatial memory impairment observed in our study may imply that the reduction of GSK3β levels in the healthy DG does not negatively impact spatial memory or the level of viral-mediated GSK3β silencing in our study is insufficient to elicit spatial memory impairment. Previous lesion studies have shown that spatial memory remains intact even when only a small portion of the dorsal hippocampus is left unlesioned (Moser et al., 1995).

Two forms of synaptic plasticity, LTP and long term depression (LTD) are generally accepted models of information storage within the hippocampus and have been considered as cellular correlates of learning and memory (Bliss and Collingridge, 1993; Bear and Abraham, 1996). LTP can be further divided into two phases; early LTP is associated with changes in trafficking and conductance of receptors at the surface of synapses while late LTP is associated with protein expression and synaptic remodeling (Abraham and Williams, 2008). GSK3β has previously been found to be implicated in cellular processes related to synaptic plasticity such as α-amino-3-hydroxy-5-methyl-4-isoxazolepropionic acid receptor (AMPAR) trafficking (Du et al., 2010; Wei et al., 2010; Xie et al., 2011; Nelson et al., 2013), N-methyl-D-aspartate receptor (NMDAR) trafficking (Chen et al., 2007; Zhu et al., 2007; Peineau et al., 2009), GABA_A_ surface receptor expression (Rui et al., 2013), pre-synaptic vesicle trafficking (Zhu et al., 2007, 2010), transcription of immediate early genes (IEGs; Graef et al., 1999), and transcription of genes required for L-LTP (Ma et al., 2011). Previous recordings from the CA1 region of the hippocampus have shown that GSK3β activity is decreased during the induction of LTP and increased during the induction of LTD (Hooper et al., 2007; Peineau et al., 2007). Subsequent pharmacological and genetic manipulations have shown that regulation of GSK3β activity is pivotal for the switch between LTP and LTD. Overactive GSK3β prevents the induction of LTP (Hooper et al., 2007; Zhu et al., 2007; Dewachter et al., 2009), while inhibition of GSK3β prevents the induction of LTD but allows LTP induction (Peineau et al., 2007, 2009; Xie et al., 2011). Induction of LTP has also been shown to inhibit GSK3β activity and prevent subsequent induction of LTD for up to an hour (Peineau et al., 2007). Our finding that long term GSK3β silencing enables induction of persistently higher LTP supports the findings of a study which showed that pharmacological inhibition of GSK3β with 10 mM LiCl for 60 min prior to high frequency stimulation (HFS) is able to enhance induction of LTP in the CA1 region (Cai et al., 2008). However, another study involving pharmacological inhibition of GSK3β with 20 mM LiCl or 2 μM CT99021 for 30 min prior to HFS showed no increase in DG-LTP in wild type animals (Franklin et al., 2014). It is possible that the shorter incubation period prior to HFS may not be sufficient to elicit a difference in DG-LTP for wild type animals.

The enhancement of LTP in the DG has been shown to play functionally differing roles from the CA1 region and can result in memory impairments (Okada et al., 2003). Our observation that LTP is enhanced in the DG of GSK3β silenced mice may therefore potentially correlate with the contextual fear memory deficits observed. This enhanced LTP but decreased in memory retrieval is consistent with studies reporting dissociation in hippocampal LTP and associative learning in mice (Sahún et al., 2007; Gruart et al., 2012). Although hippocampus is involved in associative learning, the contribution of different synapses is still poorly understood. Recent findings that reported differences in the functional aspects of different synapses within the hippocampus suggested that evolution of the timed changes in synaptic strength during functional organization did not coincide with the sequential distribution with respect to anatomical criteria and connectivity (Gruart et al., 2014). Moreover, hippocampal intrinsic and extrinsic circuits are involved in acquisition of cue and context information during associative learning (Carretero-Guillén et al., 2015). Future studies can explore the effects of GSK3β silencing on cellular processes related to synaptic efficiency in specific cell population such as adult DG granule cells.

Administration of GSK3β inhibitors to healthy individuals have resulted in impairments in cognitive performance (Weingartner et al., 1985; Stip et al., 2000; Wingo et al., 2009). We believe a targeted approach will decrease potential detrimental side effects when GSK3β activity is manipulated for therapy or prophylaxis. Our study is the first step in such a direction and future work will involve manipulating GSK3β activity in different brain regions at different developmental stages to better understand the role of GSK3β in learning and memory.

## Author Contributions

BC designed and performed all *in vitro* and *in vivo* experiments, all animal behavior test, analyzed data and wrote the manuscript; JRR tested efficiency of shRNA using Western blotting analysis; TN injected animals for some *in vivo* experiments; MD produced viruses, injected animals and did imaging for some *in vivo* experiments; JZ and ZZ provided materials for the experiments and provided critical inputs to the design of some experiments; ZB performed and analyzed data for Morris water maze experiments and provided critical inputs on animal behavioral studies; AD, SHN and SS performed and analyzed data for LTP recordings; ELKG initiated and directed the entire study, designed experiments, analyzed data and wrote the manuscript.

## Conflict of Interest Statement

The authors declare that the research was conducted in the absence of any commercial or financial relationships that could be construed as a potential conflict of interest.
